# Cognitive outcomes at ages seven and nine years in South African children from the children with HIV early antiretroviral (CHER) trial: a longitudinal investigation

**DOI:** 10.1002/jia2.25734

**Published:** 2021-07-14

**Authors:** Kaylee S van Wyhe, Barbara Laughton, Mark F Cotton, Ernesta M Meintjes, Andre JW van der Kouwe, Michael J Boivin, Martin Kidd, Kevin GF Thomas

**Affiliations:** ^1^ ACSENT Laboratory Department of Psychology University of Cape Town Cape Town South Africa; ^2^ Family Centre for Research with Ubuntu Department of Paediatrics and Child Health Faculty of Medicine and Health Sciences Stellenbosch University Cape Town South Africa; ^3^ Biomedical Engineering Research Centre Division of Biomedical Engineering Department of Human Biology University of Cape Town South Africa; ^4^ Athinoula A. Martinos Center for Biomedical Imaging Massachusetts General Hospital Charlestown MA USA; ^5^ Department of Psychiatry and Neurology and Ophthalmology Michigan State University East Lansing MI USA; ^6^ Department of Psychiatry University of Michigan Ann Arbor MI USA; ^7^ Centre of Statistical Consultation Stellenbosch University Cape Town South Africa

**Keywords:** antiretroviral therapy, The Children with HIV Early Antiretroviral Therapy (CHER) trial, HIV/AIDS, HIV‐associated neurocognitive impairment, paediatric HIV, working memory

## Abstract

**Introduction:**

Many children living with HIV (CLWH) display impaired cognition. Although early combination antiretroviral therapy (ART) produces improved cognitive outcomes, more long‐term outcome data are needed. After concluding the Children with HIV Early antiRetroviral (CHER) trial in 2011, we investigated cognitive performance, at seven and nine years of age. Participants had been randomized to deferred ART (ART‐Def; n = 22); immediate time‐limited ART for 40 weeks (ART‐40W; n = 30) and immediate time‐limited ART for 96 weeks (ART‐96W; n = 18). We also recruited HIV‐exposed uninfected (CHEU; n = 28) and HIV‐unexposed (CHU; n = 35) children.

**Methods:**

Data were collected between May 2012 and December 2017. Mixed‐model repeated‐measures ANOVAs assessed differences over time between CLWH (ART‐40W, ART‐96W and ART‐Def) and CHIV‐ CHEU and CHU between ART‐Early (ART‐40W and ART‐96W), ART‐Def, CHEU and CHU; and between ART‐40W, ART‐96W, ART‐Def, CHEU and CHU.

**Results:**

All comparisons found significant effects of Time for most outcome variables (better scores at nine than at seven years; *p*s < 0.05). The first ANOVAs found that for (a) motor dexterity, CLWH performed worse than CHIV‐ at seven years (*p* < 0.001) but improved to equivalence at nine years, (b) visual‐spatial processing and problem solving, only CLWH (*p* < 0.04) showed significant performance improvement over time and (c) working memory and executive function, CLWH performed worse than CHIV‐ at both seven and nine years (*p* = 0.03 and 0.04). The second ANOVAs found that for (a) working memory, CHU performed better than ART‐Early and CHEU (*p *< 0.01 and <0.04), and (b) motor dexterity, ART‐Def performed worse than ART‐Early, CHEU and CHU at seven years (*p* = 0.02, <0.001 and <0.001 respectively) but improved to equivalence at nine years (*p*s > 0.17). Similarly, for motor dexterity, ART‐Def performed worse than ART‐96W, CHEU and CHU at seven years (*p* < 0.04, <0.001 and <0.001) but improved to equivalence at nine years (*p*s > 0.20).

**Conclusions:**

Although neurocognitive developmental trajectories for treatment groups and controls were largely similar (i.e. performance improvements from 7 to 9), all ART‐treated children, regardless of treatment arm, remain at risk for cognitive deficits over early school ages. Although the nature of these deficits may change as cognitive development proceeds, there are potential negative consequences for these children’s future learning, reasoning and adaptive functioning.

## 1 Introduction

Despite treatment advances, many children living with HIV (CLWH) have impaired cognitive performance [1, 2, 3]. Early and severe HIV‐related neurological manifestations such as encephalopathy, increase the risk of ongoing cognitive deficits due to altered brain morphology [4, 5]. Because these deficits cannot be rehabilitated completely, infancy provides a short intervention window to improve long‐term cognitive development [6, 7].

Research into the efficacy of early intervention strategies must go beyond cross‐sectional between‐group comparisons and instead investigate long‐term effects of combination antiretroviral therapy (ART) on the developing child’s cognitive abilities [8]. This longitudinal study investigated effects on cognitive performance, at seven and nine years of age, of three intervention strategies within the Children with HIV Early antiRetroviral **(**CHER) trial.

This trial was conducted in South Africa from 2005 to 2011 [9, 10]. Asymptomatic CLWH infants (N = 377; CD4 cell count ≥25% at a mean age of seven weeks) were randomized to either immediate (i.e. before 12 weeks of age) ART initiation and planned interruption 40 weeks later (ART‐40W); immediate ART initiation and planned interruption at 96 weeks (ART‐96W); or deferred ART initiation until clinical or immunological disease progression (ART‐Def), in line with the World Health Organization (WHO) 2006 ART criteria.

The CHER trial found that early ART initiation significantly reduced mortality and HIV disease progression [9]. However, HIV may continue to affect the brain and body throughout critical cognitive developmental stages of childhood and adolescence [11, 12].

A CHER neurodevelopmental sub‐study investigated participants at a mean age of 11 months [13], those in the early treatment groups (n = 64) had significantly better gross motor and global scores than those in the ART‐Def group (n = 26) on the Griffiths Mental Development Scales [14], suggesting benefits of early ART initiation. By five years of age, there were no significant differences between ART‐40W, ART‐96W, ART‐Def and HIV‐uninfected control groups. However, all CLWH groups had poorer visual perception [15].

In another randomized study, the Pediatric Randomized Early versus Deferred Initiation in Cambodia and Thailand (PREDICT) trial [16] 299 children, aged one to twelve years and with CD4 percentage between of 15%–24% and no AIDS‐defining illness, to either immediate (n = 149) or deferred (n = 150) ART. Three‐year follow‐up revealed no notable benefits of earlier treatment. A neurodevelopmental sub‐study found no differences in cognitive performance at a mean age of nine years, but both groups performed worse than uninfected children on tests of general intellectual functioning, visual‐motor integration and memory [17].

### 1.1 The current study

We investigated cognition at seven and nine years of age in children from the CHER neurodevelopmental sub‐study [15]. Our study population comprised the three HIV+ groups from the CHER trial: early ART until week 40 (ART‐40W), or until week 96 (ART‐96W) followed by planned interruption; and ART‐Def, with delayed but continuous ART. ART‐40W and ART‐96W re‐started treatment after interruption according to clinical or immunological criteria [9]. In addition, three children with baseline CD4 <25% were included in ART‐40W and one in ART‐96W. Comparison groups comprised of children HIV‐exposed uninfected (CHEU) and HIV‐unexposed (CHU). We used a repeated‐measures factorial design to test hypotheses related to (a) effects of HIV (i.e. we expected that HIV‐uninfected participants would perform better at both seven and nine years than CLWH); (b) effects of early time‐limited intervention (e.g. we expected that ART‐40W and ART‐96W participants would perform better at both measurement points than ART‐Def participants) and (c) developmental trajectory (i.e. we expected that all participants would show improvements in cognitive performance from seven years to nine years). The design also allowed us to investigate whether trajectories of cognitive development between seven and nine years were different in HIV‐uninfected versus CLWH, and in immediate versus deferred treatment groups.

## 2 Methods

### 2.1 Research design and setting

We used an observational‐longitudinal design. Data were collected between May 2012 and December 2017. CLWH had regular three‐monthly adherence visits and clinical evaluations. HIV‐uninfected participants had six‐monthly clinical assessments.

### 2.2 Participants

At the end of the CHER trial in 2011, CLWH from Cape Town were enrolled in a follow‐up longitudinal neuroimaging and cognitive study [10]. HIV‐uninfected controls included those enrolled in the earlier neurodevelopmental sub‐study [15], with additional children recruited from similar neighbourhoods after attrition within the original control group. Inclusion criteria were: willingness of caregivers/legal guardians to participate; documented evidence of child and caregiver’s HIV status; child’s birth weight >2000 g; appropriate age of child participants; and child’s home language either English, Xhosa or Afrikaans. Exclusion criteria included: history of medical or psychiatric disorder that might affect cognitive performance; history of head injuries or birth complications; pervasive neurological, developmental or learning disability; and use of any psychiatric medication except stimulants to treat attention‐deficit/hyperactivity disorder (ADHD).

**Figure 1:**
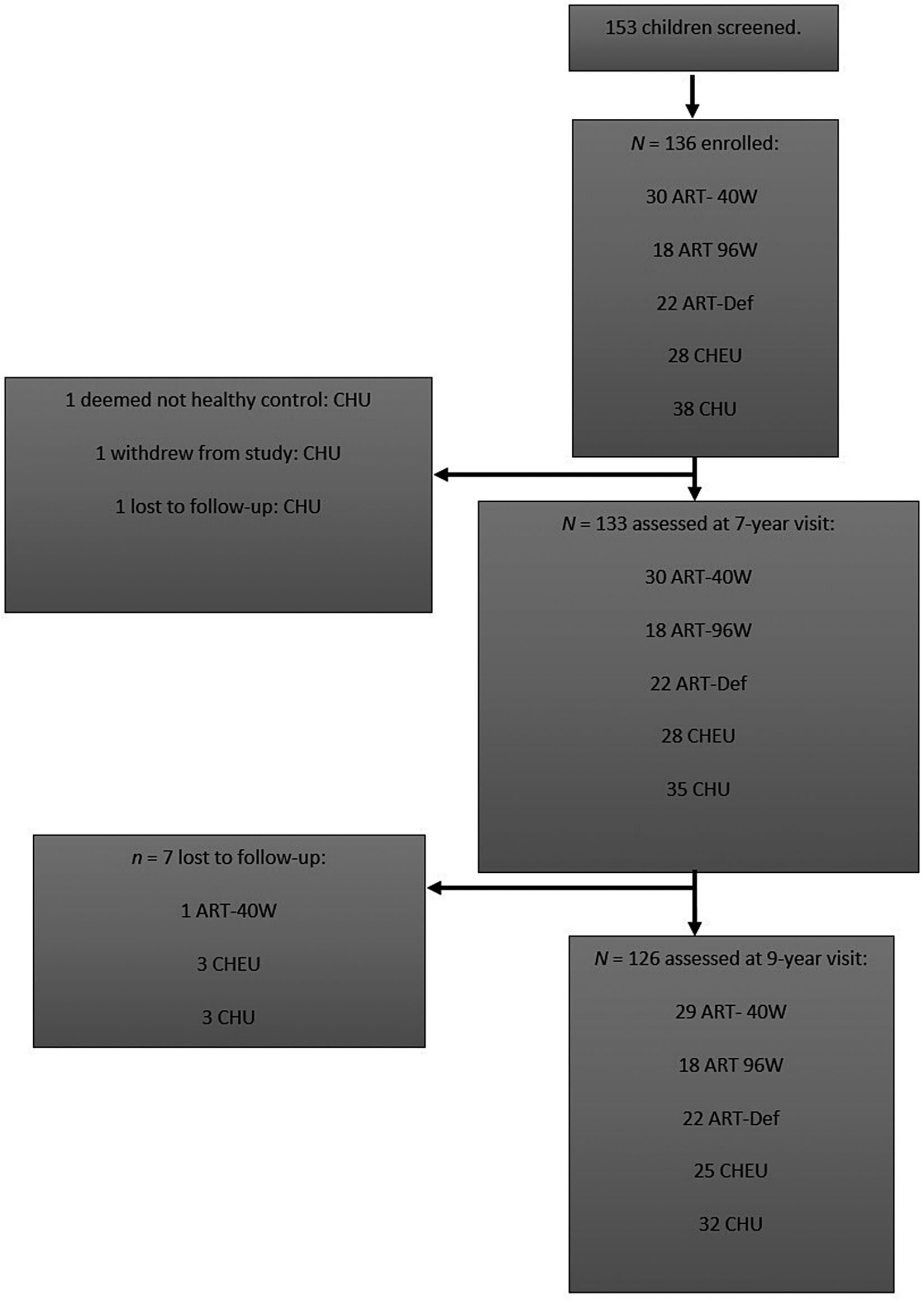
Participant flow and attrition at each stage of the study procedures. The seven‐year visits took place between May 2012 and December 2014. The nine‐year visits took place between May 2014 and December 2017.

Figure 1 shows the flow and attrition of participants throughout the study. The final samples for data analysis were collected from 133 children at age seven years and 126 children at age nine years.

### 2.3 Procedures and measures

#### 2.3.1 Neuropsychological assessment

Participants were administered a comprehensive test battery at age seven years and nine years by trained research assistants supervised by a licensed psychologist. Test stimuli and instructions were presented in the participant’s preferred language (English, Xhosa or Afrikaans). Rigorous translation and back‐translation (conducted under license from test publishers) ensured that different versions of the instruments were of equivalent complexity. Assessments were video recorded for quality control.

The battery consisted of standardized tests, commonly used in paediatric neuropsychological assessment, with strong psychometric properties and proven utility across different cultural and language settings [1, 13, 15, 18, 19, 20, 21]. It included the Purdue Pegboard Test [PPT; 22], a measure of fine‐motor coordination (assessment of preferred hand only); the Beery‐Buktenika Developmental Test of Visual‐Motor Integration [Beery‐VMI; 23], which assesses visual perception and motor‐coordination integration ability; a category fluency (animal naming) test that assesses verbal generativity, a component of executive functioning [24]; the Kaufman Assessment Battery for Children – Second Edition [KABC‐II; 25]; the Test of Variables of Attention [TOVA; 26], a computerized test often used for diagnosis and monitoring of children with attention deficits and the Peabody Picture Vocabulary Test – fourth Edition [PPVT‐IV; 27], a measure of receptive language ability.

Regarding the KABC‐II, we administered 13 subtests to derive indices termed Sequential Processing (an assessment of auditory working memory), Simultaneous Processing (visual‐spatial processing and problem solving), Learning (short‐term memory) and Planning (executive reasoning). Aggregating performance on KABC‐II subtests not relying on verbal expression allows derivation of a Nonverbal Index (NVI). Summing across all KABC‐II subtests allows derivation of a Mental Processing Index (MPI; an estimate of the child’s overall cognitive performance). Regarding the TOVA, the primary outcome variable used was the ADHD index score. High scores indicate better functioning (i.e. scores closer to zero on the negative end are better).

#### 2.3.2 Other data collection

To collect biographic, economic and medical information, a study clinician administered study‐specific questionnaire to the parent/guardian. To measure ART adherence, we used pill count data from the clinic visit closest to each of the test sessions.

### 2.4 Ethical considerations

Research ethics committees at the Universities of Cape Town and Stellenbosch approved the study, which adhered to the principles of the Declaration of Helsinki [28]. The consent process was conducted in person and in the participant’s preferred language. A parent or legal guardian provided consent for the child’s participation; child participants provided assent at nine years.

### 2.5 Data management and statistical analysis

Analyses were conducted using Statistica (version 13). All reported *p*‐values are two‐tailed, with *α* set at 0.05 for decisions regarding statistical significance. We examined distributional assumptions underlying parametric statistical tests for all variables of interest and adjusted analytic plans where necessary. For the Beery‐VMI, KABC‐II and TOVA we used age‐adjusted standardized scores based on US norms. For the PPT, category fluency test and PPVT‐IV we used raw scores.

#### 2.5.1 Inferential analyses

First, chi‐squared tests of contingency, Fisher’s exact tests, one‐way ANOVAs or Kruskal–Wallis tests examined between‐group differences for sample sociodemographic and clinical characteristics. Second, a series of two‐way mixed‐model repeated‐measures ANOVAs, with group assignment and age (seven and nine years) included as fixed effects, participants (nested in groups) included as a random effect, and performance on a cognitive variable as the outcome, allowed simultaneous examination of between‐group differences at each measurement point and change over time. Due to the small sample size and to fully understand the impact of HIV on the developing brain, the first set of models compared CLWH (combined ART‐40W, ART‐96W and ART‐Def) to CHIV‐ (combined CHEU and CHU). The second set compared an ART‐Early group (combined ART‐40W and ART‐96W groups) to ART‐Def, CHEU and CHU groups. The third set compared the five groups (ART‐40W, ART‐96W, ART‐Def, CHEU, CHU) against one another. We used Fisher’s Least Significant Difference tests for pairwise post hoc comparisons. For each ANOVA, we calculated effect size estimates (i.e. eta squared or partial eta squared), which capture the magnitude of the difference in the population. We interpreted effect sizes as being small (0.01), medium (0.06) or large (0.14), by convention [29]. Finally, because there were significant between‐group differences in home language, we used a series of ANOVAs to explore its influence on cognitive performance.

## 3 Results

### 3.1 Sociodemographic characteristics

Most child participants (at least 75% in each of the five groups) were raised by their biological mothers. Among both children and mothers, there were no significant between‐group differences in education, with 76% of the child participants in age‐appropriate grades. Relatively few mothers were high school graduates, but most had completed ≥10 years of formal education. All participants were from low socioeconomic status (SES) backgrounds, with similar household income across groups. There was a significant between‐group difference for home language distribution: In the CHU group 37% of participants spoke Afrikaans and the rest Xhosa, whereas in the other groups almost all children spoke Xhosa (Table 1).

**Table 1:** Sample sociodemographic characteristics (N = 133)

Variable	ART‐40W	ART‐96W	ART‐Def	CHEU	CHU	*F*/*χ* ^2^	*p*	ESE
	(n = 30)	(n = 18)	(n = 22)	(n = 28)	(n = 35)			
Mother as primary caregiver	25 (85.29)	12 (75.00)	15 (78.95)	26 (92.86)	30 (90.91)	4.23	0.38	0.19
Mother’s education level								
<Grade 10	7 (19.4)	9 (34.61)	2 (9.09)	5 (17.85)	8 (22.85)	0.78	0.54	0.03
Completed Grade 10	11 (30.55)	7 (26.92)	8 (36.36)	10 (35.17)	17 (48.57)			
Completed Grade 12	5 (13.88)	1 (3.84)	3 (13.63)	5 (17.85)	1 (2.85)			
Post‐Grade 12 diploma	0 (0)	1 (3.84)	1 (4.54)	2 (7.14)	3 (8.57)			
Household income (ZAR)^a^	2062 (330.72)	1750 (612.37)	2131 (376.09)	2008 (458.87)	1917 (601.64)	1.71	0.14	0.05
Home language (Xhosa:Afrikaans:English)	25:4:1	14:4:0	21:1:0	25:1:2	22:13:0	21.71	0.01**	0.37
Child’s sex (M:F)	15:15	7:11	8:14	17:11	19:6	4.10	0.39	0.17
Age‐appropriate grade at 9‐year visit (%)^b^	79.31	83.33	72.73	64.00	81.25	3.19	0.53	16
Child’s duration of education (months)								
7‐year measurement point	20.13 (3.73)	19.38 (6.06)	21.27 (3.86)	20.69 (5.26)	20.85 (6.28)	0.41	0.93	0.01
9‐year measurement point	43.27 (3.54)	44.16 (5.60)	45.45 (4.67)	43.12 (5.44)	44.46 (5.9)	0.63	0.64	0.02

Data are raw numbers, with percentages in parentheses except for household income and child’s duration of education we report means and standard deviation in parentheses. Unless otherwise specified, data refer to measures taken at study enrolment. ART‐40W, ART 40 weeks (treatment started early with planned interruption at 40 weeks); ART‐96W, ART 96 weeks (treatment started early with planned interruption at 96 weeks); ART‐Def, ART‐Deferred (treatment deferred until clinical or immunological disease progression was evident); CHEU, children HIV‐exposed uninfected; CHU, children HIV‐unexposed; ESE, effect size estimate ($n_{p}^{2}$).

^a^ Over the period of enrolment, the average exchange rate was US$: ZAR = 8.65

^b^ government policy states that South African children should have their ninth birthday in Grade 3 [30]. Hence, those participants who were in an age‐appropriate grade at their nine‐year visit were all either in Grade 3 or Grade 4.

* *p* < .05

** *p* < .01

*** *p* < .001.

### 3.2 Sample physical and clinical characteristics

Analyses of both the seven‐ and nine‐year data detected no significant between‐group differences for viral load, CD4 percent, ART compliance (close to 100% for all CLWH), weight and height (see Table 2).

**Table 2:** Sample physical and clinical characteristics (N = 133)

Variable	ART‐40W	ART‐96W	ART‐DEF	CHEU	CHU	*F*/*χ* ^2^	*p*	ESE
	(n = 30)	(n = 18)	(n = 22)	(n = 28)	(n = 35)			
Viral load suppressed %^a^								
7 years	92.31	93.75	86.36	–	–	0.72	0.69	0.11
9 years	92.59	86.67	90.48	–	–	0.38	0.82	0.08
CD4 count %								
7 years	39.28	36.41	37.93	–	–	0.43	0.65	0.01
9 years	39.77	35.78	39.46	–	–	1.48	0.49	0.03
ART Drug 1 compliance %								
7 years	99.17 (1.85)	99.23 (1.14)	97.95 (6.11)	–	–	0.67	0.52	0.02
9 years	99.85 (0.44)	98.94 (1.78)	98.68 (2.69)	–	–	2.25	0.11	0.07
ART Drug 2 compliance %								
7 years	98.48 (2.45)	99.00 (1.90)	99.71 (0.56)	–	–	2.78	0.07	0.08
9 years	99.79 (0.62)	98.88 (2.17)	98.86 (2.07)	–	–	1.85	0.16	0.06
ART Drug 3 compliance %								
7 years	99.03 (1.90)	98.57 (2.73)	99.35 (1.27)	–	–	1.32	0.27	0.05
9 years	98.36 (4.78)	98.50 (1.29)	100.00 (0.00)	–	–	0.51	0.60	0.04
Weight (*z*‐scores)^b^								
7 years	0.09 (0.99)	−0.13 (0.73)	0.05 (1.09)	0.35 (1.23)	0.21 (1.49)	2.21	0.69	–
9 years	−0.10 (1.25)	−0.34 (0.80)	−0.12 (1.30)	0.03 (1.35)	0.27 (1.36)	1.99	0.73	–
Height (*z*‐scores)^c^								
7 years	−0.44 (0.96)	−0.22 (0.98)	−0.30 (0.98)	0.05 (1.18)	0.06 (1.21)	3.83	0.43	–
9 years	−0.60 (1.16)	−0.50 (0.90)	−0.66 (0.99)	−0.35 (1.22)	0.002 (1.26)	4.76	0.31	–

The variable *ART Drug Compliance* refers to participants’ adherence to each antiretroviral therapy tablet. ART‐40W, ART 40 weeks (treatment started early with planned interruption at 40 weeks); ART‐96W, ART 96 weeks (treatment started early with planned interruption at 96 weeks); ART‐Def, ART‐Deferred (treatment deferred until clinical or immunological disease progression was evident); CHEU, children HIV‐exposed and ‐uninfected; CHU, children HIV‐unexposed; ESE, effect size estimate ($n_{p}^{2}$).

^a^ The lower limit of viral detection was <39 units

^b^ World Health Organization (WHO) weight‐for‐age *z*‐score mean (SD)

^c^ WHO height‐for‐age *z*‐score mean (SD).

During the CHER trial, most CLWH (81.7%) were asymptomatic. At five years of age in the neurodevelopmental sub‐study, 28 (35.9%) of 78 CLWH had either Centers for Disease Control severe stage B or C disease, 21 (30%) are included in this study. The most common diagnoses were HIV encephalopathy (HIVE), failure to thrive, oesophageal candidiasis and HIV wasting syndrome. All CLWH had been initiated on a first‐line ART (zidovudine/lamivudine/lopinavir‐ritonavir). Sixty‐two of the 69 CLWH (89.85%) were still on the original first line ART regimen at the nine‐year assessment. Overall, 13 children had HIVE (ART‐40W n = 5, ART‐96W n = 3, ART‐DEF n = 5); 11 have since recovered [31].

### 3.3 Cognitive performance

#### 3.3.1 Comparison of CLWH and CHIV‐ at seven and nine years

Analyses detected a significant main effect of Time, with higher scores at nine years than at seven years, for most outcome variables (see Tables 3 and 4). However, the Beery‐VMI and KABC‐II Sequential Processing Index (significantly higher scores at seven years than at nine years) and the TOVA (no significant main effect of Time).

**Table 3:** Cognitive performance in children living with HIV (Early and Deferred Treatment) and HIV‐uninfected children at seven and nine years, Part I (N = 133)

Outcome variable	Group						
	CLWH	CHIV‐				95% CI	
	(n = 70)	(n = 63)	*F*	*p*	ESE	LL	UL
Beery‐VMI							
7 years	92.63 (8.86)	92.64 (9.26)					
9 years	85.69 (8.90)	86.70 (10.17)					
Time			38.95	<0.001***	0.18	81.20	96.60
Group			0.04	0.85	<0.01	84.40	93.40
Time×Group			1.73	0.19	<0.01	79.90	98.60
Semantic fluency^a^							
7 years	5.62 (2.38)	6.32 (2.53)					
9 years	7.73 (3.00)	9.07 (2.88)					
Time			89.31	<0.001***	0.33	5.50	10.50
Group			4.73	0.03*	0.02	6.10	9.80
Time×Group			1.50	0.22	<0.01	4.55	11.70
PPT^a^							
7 years	9.01 (1.79)	9.77 (1.66)					
9 years	12.00 (1.43)	11.62 (1.60)					
Time			177.36	<0.001***	0.45	8.55	13.05
Group			0.59	0.45	<0.01	9.92	11.53
Time×Group			10.67	<0.01**	0.03	8.00	13.00
PPVT‐IV^a^							
7 years	55.63 (14.31)	59.94 (16.11)					
9 years	78.74 (23.26)	85.91 (22.38)					
Time			193.95	<0.001***	0.50	60.00	110.00
Group			2.15	0.14	<0.01	73.80	96.35
Time×Group			0.32	0.58	<0.01	60.00	110.00
TOVA							
7 years	−0.22 (2.27)	−0.21 (2.48)					
9 years	−0.86 (3.13)	−0.68 (2.98)					
Time			4.07	0.05	0.02	−2.03	0.87
Group			0.00	0.94	<0.01	−1.92	0.72
Time×Group			0.28	0.60	<0.01	−2.31	1.32

Data in columns 2‐3 are M (SD). CLWH, children living with HIV; CHIV‐, HIV‐uninfected children; ESE, effect size estimate (n^2^); Beery‐VMI, Beery‐Buktenika Developmental Test of Visual‐Motor Integration; PPT, Purdue Pegboard Test; PPVT‐IV, Peabody Picture Vocabulary Test – fourth Edition; TOVA, Test of Variables of Attention.

^a^ Raw scores reported.

* *p* < .05

** *p* < .01

*** *p* < .001.

**Table 4:** Cognitive performance in children living with HIV (Early and Deferred Treatment) and HIV‐uninfected children at seven and nine years, Part II (N = 133)

KABC‐II Index	Group						
	CLWH	CHIV‐				95% CI	
	(n = 70)	(n = 63)	*F*	*p*	ESE	LL	UL
Sequential processing							
7 years	83.60 (9.07)	85.48 (11.84)					
9 years	79.22 (7.77)	84.07 (11.82)					
Time			17.90	<0.001***	0.10	80.40	93.60
Group			4.25	0.04*	0.02	80.00	92.50
Time×Group			3.54	0.06	0.02	76.50	95.20
Simultaneous processing							
7 years	78.70 (10.37)	81.61 (12.15)					
9 years	82.00 (13.94)	79.53 (13.19)					
Time			0.23	0.63	<0.01	76.65	88.20
Group			0.09	0.76	<0.01	76.16	88.48
Time×Group			5.12	0.03*	0.03	75.00	90.00
Learning ability							
7 years	75.88 (9.80)	78.48 (11.44)					
9 years	78.93 (11.29)	81.84 (13.41)					
Time			9.24	<0.001***	0.06	75.00	88.75
Group			1.48	0.23	<0.01	74.25	89.10
Time×Group			0.06	0.80	<0.01	72.00	89.60
Planning ability							
7 years	72.65 (8.82)	74.63 (9.64)					
9 years	75.88 (8.49)	76.07 (12.76)					
Time			6.54	0.01*	0.04	73.77	84.77
Group			0.15	0.70	<0.01	73.88	83.77
Time×Group			1.24	0.27	<0.01	72.50	85.00
Non‐verbal reasoning							
7 years	72.81 (10.08)	74.60 (11.15) (11.03)					
9 years	76.17 (10.65)	75.71 (14.13)					
Time			4.44	0.04*	0.02	72.45	85.10
Group			0.00	0.97	<0.01	73.07	84.08
Time×Group			1.25	0.27	<0.01	71.05	87.00
Mental processing Index (Std scores)							
7 years	71.90 (7.32)	74.59 (9.70)					
9 years	73.01 (6.77)	74.71 (11.69)					
Time			0.91	0.34	<0.01	72.21	81.34
Group			0.92	0.34	<0.01	72.00	82.00
Time×Group			0.65	0.42	<0.01	71.02	82.68

Data in columns 2‐3 are M (SD). CLWH, children living with HIV; CHIV‐, HIV‐uninfected children; ESE, effect size estimate (n^2^); KABC‐II, Kaufman Assessment Battery for Children – Second Edition.

* *p* < .05

** *p* < .01

*** *p* < .001.

There were also two significant main effects of Group: for executive function (semantic fluency raw score) and auditory working memory (KABC‐II Sequential Processing Index score), CLWH performed worse than CHIV‐ (*p* = 0.03 and 0.04 respectively). Finally, the analyses detected two significant Time by Group interaction effects: for fine motor dexterity (PPT raw score), CLWH performed worse than CHIV‐ at seven years (*p* < 0.001) but improved to equivalence at nine years. For visual‐spatial processing and problem solving (KABC‐II Simultaneous Processing Index score), CLWH (*p* = 0.03) but not CHIV‐ showed significant performance improvement over time.

#### 3.3.2 Comparison of ART‐early, ART‐Def, CHEU and CHU at seven and nine years

Analyses detected a significant main effect of Time, with higher scores at nine years than at seven years, for most outcome variables (see Tables 5 and 6). Again, the exceptions here were the Beery‐VMI, TOVA and KABC‐II Sequential Processing Index, where analyses detected significantly higher scores at seven years than at nine years.

**Table 5:** Cognitive performance in children with living with HIV with Early ART initiation, children living with HIV with deferred ART and HIV‐uninfected children at seven and nine years, Part I (N = 133)

Outcome variable	Group								
	ART‐Early	ART‐Def	CHEU	CHU				95% CI	
	(n = 48)	(n = 22)	(n = 28)	(n = 35)	*F*	*p*	ESE	LL	UL
Beery‐VMI									
7 years	92.80 (9.18)	92.27 (9.64)	92.44 (8.08)	90.29 (9.40)					
9 years	86.34 (9.28)	84.24 (8.01)	85.04 (10.1)	88.29 (10.10)					
Time					40.25	<0.001***	0.18	81.20	96.60
Group					0.18	0.91	<0.01	82.50	94.60
Time×Group					1.69	0.17	0.02	77.70	98.70
Semantic fluency^a^									
7 years	5.53 (2.50)	5.82 (2.13)	6.15 (2.44)	6.47 (2.63)					
9 years	7.97 (3.16)	7.19 (2.60)	9.16 (2.83)	9.00 (2.96)					
Time					78.66	<0.001***	0.31	5.50	10.50
Group					1.61	0.19	0.02	5.46	10.08
Time×Group					1.47	0.23	0.02	4.20	11.90
PPT^a^									
7 years	9.31 (1.72)	8.36 (1.81)	9.89 (1.72)	9.69 (1.64)					
9 years	12.04 (1.59)	11.91 (1.06)	11.36 (1.55)	11.80 (1.64)					
Time					174.91	<0.001***	0.44	8.55	13.05
Group					1.30	0.28	<0.01	9.20	11.73
Time×Group					4.99	<0.001***	0.04	7.15	13.65
PPVT‐IV^a^									
7 years	54.45 (15.52)	58.09 (11.32)	59.53 (17.31)	60.26 (15.33)					
9 years	79.51 (22.62)	77.09 (25.04)	86.28 (28.61)	86.60 (22.84)					
Time					173.25	<0.001***	0.47	63.00	108.00
Group					0.92	0.43	<0.01	72.50	100.00
Time×Group					0.63	0.60	<0.01	55.00	115.50
TOVA									
7 years	−0.23 (2.25)	−0.19 (2.37)	−0.32 (2.48)	−0.14 (2.52)					
9 years	−0.80 (3.25)	−0.99 (2.92)	−1.07 (2.87)	−0.32 (3.08)					
Time					4.12	0.04*	0.03	−2.03	1.16
Group					0.27	0.85	<0.01	−2.38	1.36
Time×Group					0.24	0.87	<0.01	−3.01	1.72

Data in columns 2‐5 are M (SD). ART‐Early, treatment started early with planned interruption at either 40 or 96 weeks; ART‐Def, ART‐Deferred (treatment deferred until clinical or immunological disease progression was evident); CHEU, children HIV‐exposed uninfected; CHU, children HIV‐unexposed; ESE, effect size estimate (n^2^); Beery‐VMI, Beery‐Buktenika Developmental Test of Visual‐Motor Integration; PPT, Purdue Pegboard Test; PPVT‐IV, Peabody Picture Vocabulary Test – fourth Edition; TOVA, Test of Variables of Attention.

Raw scores reported.

* *p* < 0.05

** *p* < 0.01

*** *p* < 0.001.

**Table 6:** Cognitive performance in children living with HIV with Early ART initiation, children living with HIV with deferred ART and HIV‐uninfected children at seven and nine years, Part II (N = 133)

KABC‐II index	Group								
	ART‐Def	ART‐Early	CHEU	CHU				95% CI	
	(n = 22)	(n = 48)	(n = 28)	(n = 35)	*F*	*p*	ESE	LL	UL
Sequential processing									
7 years	85.73 (7.65)	82.62 (9.59)	84.21 (12.18)	86.49 (11.65)					
9 years	78.59 (7.26)	79.51 (8.05)	82.48 (12.91)	85.31 (10.94)					
Time					200.87	<0.001***	0.11	80.00	93.75
Group					20.89	0.04*	0.04	78.20	96.90
Time×Group					10.87	0.08	0.03	73.60	98.90
Simultaneous Processng									
7 years	81.36 (9.84)	77.45 (10.49)	80.41 (12.35)	82.54 (12.09)					
9 years	85.00 (13.46)	80.60 (14.09)	77.48 (13.36)	81.23 (13.05)					
Time					00.22	0.64	<0.01	77.00	89.10
Group					10.18	0.32	0.02	74.00	94.35
Time×Group					10.78	0.15	0.03	71.30	96.60
Learning Ability									
7 years	77.18 (9.67)	75.20 (9.90)	77.33 (9.95)	81.17 (12.38)					
9 years	79.82 (9.71)	78.51 (12.03)	80.28 (13.06)	83.13 (13.77)					
Time					90.37	<0.001***	0.06	75.40	89.70
Group					10.04	0.38	0.02	74.25	92.40
Time×Group					00.58	0.63	0.01	72.00	92.00
Planning Ability									
7 years	75.09 (9.40)	71.51 (8.41)	72.64 (9.13)	76.23 (9.86)					
9 years	76.05 (9.40)	75.81 (8.14)	74.16 (11.54)	77.56 (13.63)					
Time					40.65	0.03*	0.03	74.46	84.66
Group					10.26	0.29	0.02	72.90	87.75
Time×Group					10.02	0.39	0.02	69.70	88.40
Non‐verbal REASONING									
7 years	75.68 (9.69)	71.46 (10.08) (10.03) (10.15)	71.71 (11.16) (11.03)	76.91 (10.74)					
9 years	76.73 (9.94)	75.91 (11.06)	74.52 (15.34)	76.65 (13.69)					
Time					30.46	0.07	0.02	73.08	85.84
Group					00.98	0.40	0.02	70.95	89.10
Time×Group					10.34	0.27	0.02	68.00	90.00
Mental processing Index (Std scores)									
7 years	73.95 (6.20)	70.86 (7.69)	71.43 (7.42)	76.18 (9.93)					
9 years	73.82 (5.81)	72.64 (7.20)	72.96 (11.65)	76.16 (11.72)					
Time					00.61	0.44	<0.01	72.90	81.81
Group					10.67	0.18	0.03	70.20	85.05
Time×Group					00.94	0.42	0.02	69.00	85.50

Data in columns 2‐5 are M (SD). ART‐Early, treatment started early with planned interruption at either 40 or 96 weeks; ART‐Def, ART‐Deferred (treatment deferred until clinical or immunological disease progression was evident); CHEU, children HIV‐exposed uninfected; CHU, children HIV‐unexposed; ESE, effect size estimate (n^2^); KABC‐II, Kaufman Assessment Battery for Children – Second Edition.

* *p* < 0.05

** *p* < 0.01

*** *p* < 0.001.

There was one significant main effect of Group: for auditory working memory (KABC‐II Sequential Processing Index score), CHU performed significantly better than ART‐Early and CHEU (*p* = 0.01 and 0.04 respectively) and better than ART‐Def (*p* = 0.05). Finally, there was one significant Time by Group interaction effect: for fine motor dexterity (PPT raw score), ART‐Def performed more poorly than ART‐Early, CHEU and CHU at seven years (*p* = 0.02, <0.001 and <0.001 respectively), but improved to equivalence by nine years (*p*s ≥ 0.05).

#### 3.3.3 Comparison of ART‐40W, ART‐96W, ART‐Def, CHEU and CHU at seven and nine years

Analyses detected a significant main effect of Time for most outcome variables (see Table 7 and 8). Averaged across the five groups, scores were significantly higher at nine years than at seven years for semantic fluency, M = 8.35 ± 3.00 versus 5.95 ± 2.47; PPT, M = 11.83 ± 1.52 versus 9.37 ± 1.77; PPVT‐IV, M = 81.92 ± 24.39 versus 57.70 ± 15.29; KABC‐II Learning Ability Index, M = 80.21 ± 12.31 versus 77.62 ± 10.74; KABC‐II Planning Ability Index, M = 75.97 ± 10.59 versus 73.60 ± 9.24; and KABC‐II Non‐Verbal Index, M = 75.97 ± 12.41 versus 73.66 ± 10.60. However, scores were significantly lower at nine years than at seven years for the Beery‐VMI, M = 86.15 ± 9.48 versus 91.98 ± 9.07; TOVA, M = −0.78 ± 3.05 versus −0.21 ± 2.36 and KABC‐II Sequential Processing Index, M = 81.41 ± 10.07 versus 84.50 ± 10.49.

**Table 7:** Cognitive performance in children living with HIV (three treatment arms) and HIV‐uninfected children at seven and nine years, Part I (N = 133)

Outcome variable	ART‐40W	ART‐96W	ART‐Def	CHEU	CHU					95% CI	
	(n = 30)	(n = 18)	(n = 22)	(n = 28)	(n = 35)	*F*		*p*	ESE	LL	UL	
Beery‐VMI												
7 years	92.24 (8.67)	93.72 (10.15)	92.27 (9.64)	92.44 (8.08)	90.29 (9.40)							
9 years	86.96 (8.49)	85.33 (10.60)	84.24 (8.01)	85.04 (10.1)	88.29 (10.10)							
Time						45.32		<0.001***	0.20	81.00	97.50	
Group						0.15		0.96	<0.01	82.80	96.00	
Time×Group						1.56		0.19	0.30	77.00	101.20	
Semantic fluency^a^												
7 years	5.52 (2.78)	5.55 (2.06)	5.82 (2.13)	6.15 (2.44)	6.47 (2.63)							
9 years	8.07 (3.22)	7.83 (3.14)	7.19 (2.60)	9.16 (2.83)	9.00 (2.96)							
Time						82.03		<0.001***	0.32	5.50	10.50	
Group						1.41		0.23	0.02	5.46	10.08	
Time×Group						1.13		0.35	0.02	4.20	11.90	
PPT^a^												
7 years	9.31 (1.69)	9.33 (1.81)	8.36 (1.81)	9.89 (1.72)	9.69 (1.64)							
9 years	11.79 (1.47)	12.44 (1.72)	11.91 (1.06)	11.36 (1.55)	11.80 (1.64)							
Time						194.33		<0.001***	0.47	8.55	13.05	
Group						1.09		0.31	0.01	9.36	12.22	
Time×Group						3.97		<0.001***	0.04	7.00	14.00	
PPVT‐IV^a^												
7 years	56.79 (16.27)	50.47 (13.67)	58.09 (11.32)	59.53 (17.31)	60.26 (15.33)							
9 years	83.83 (24.10)	72.55 (18.56)	77.09 (25.04)	86.28 (28.61)	86.60 (22.84)							
Time						179.07		<0.001***	0.48	63.00	108.00	
Group						1.42		0.23	0.01	64.60	98.60	
Time×Group						0.68		0.60	<0.01	52.00	117.00	
TOVA												
7 years	−0.10 (2.28)	−0.44 (2.24)	−0.19 (2.37)	−0.32 (2.48)	−0.14 (2.52)							
9 years	−0.82 (3.01)	−0.76 (3.70)	−0.99 (2.92)	−1.07 (2.87)	−0.32 (3.08)							
Time						4.17		0.04*	0.03	−2.03	1.16	
Group						0.24		0.92	<0.01	−2.45	1.40	
Time×Group						0.25		0.91	<0.01	−3.08	1.76	

Data in columns 2‐6 are M (SD). ART‐40W, ART 40 weeks (treatment started early with planned interruption at 40 weeks); ART‐96W, ART 96 weeks (treatment started early with planned interruption at 96 weeks); ART‐Def, ART‐Deferred (treatment deferred until clinical or immunological disease progression was evident); CHEU, children HIV‐exposed uninfected; CHU, children HIV‐unexposed; ESE, effect size estimate (n^2^); Beery‐VMI, Beery‐Buktenika Developmental Test of Visual‐Motor Integration; PPT, Purdue Pegboard Test; PPVT‐IV, Peabody Picture Vocabulary Test – fourth Edition; TOVA, Test of Variables of Attention.

^a^ Raw scores reported.

* *p* < 0.05

** *p* < 0.01

*** *p* < 0.001.

**Table 8:** Cognitive performance in children living with HIV (three treatment arms) and HIV‐uninfected children at seven and nine years, Part II (N = 133)

KABC‐II Index	Group									
	ART‐40W	ART‐96W	ART‐Def	CHEU	CHU				95% CI	
	(n = 30)	(n = 18)	(n = 22)	(n = 28)	(n = 35)	*F*	*p*	ESE	LL	UL
Sequential processing										
7 years	82.96 (9.89)	82.05 (9.33)	85.73 (7.65)	84.21 (12.18)	86.49 (11.65)					
9 years	79.24 (8.66)	79.94 (7.18)	78.59 (7.26)	82.48 (12.91)	85.31 (10.94)					
Time						19.88	<0.001***	0.10	80.00	92.50
Group						1.23	0.30	0.04	78.20	96.90
Time×Group						1.87	0.12	0.04	73.60	98.90
Simultaneous processing										
7 years	77.93 (9.31)	76.66 (12.40)	81.36 (9.84)	80.41 (12.35)	82.54 (12.09)					
9 years	80.00 (14.10)	81.56 (14.40)	85.00 (13.46)	77.48 (13.36)	81.23 (13.05)					
Time						0.99	0.32	<0.01	76.56	89.32
Group						0.86	0.49	0.02	73.50	94.50
Time×Group						1.53	0.20	0.03	67.60	96.20
Learning ability										
7 years	75.93 (11.77)	74.00 (5.68)	77.18 (9.67)	77.33 (9.95)	81.17 (12.38)					
9 years	79.06 (14.22)	77.61 (7.59)	79.82 (9.71)	80.28 (13.06)	83.13 (13.77)					
Time						10.16	<0.001***	0.07	74.10	88.40
Group						0.87	0.49	0.02	70.00	92.00
Time×Group						0.48	0.75	0.01	69.00	92.00
Planning ability										
7 years	69.44 (8.10)	73.83 (8.01)	75.09 (9.40)	72.64 (9.13)	76.23 (9.86)					
9 years	73.86 (5.64)	78.94 (10.48)	76.05 (9.40)	74.16 (11.54)	77.56 (13.63)					
Time						7.31	<0.001***	0.04	73.50	85.05
Group						1.98	0.10	0.05	69.70	88.40
Time×Group						0.76	0.55	0.02	66.70	92.00
Non‐verbal reasoning										
7 years	70.48 (9.50) (10.03) ((10.15)	73.05 (11.03)	75.68 (9.69)	71.71 (11.16) (11.03)	76.91 (10.74)					
9 years	74.13 (7.95)	78.77 (14.59) (13.99)(13.99)	76.73 (9.94)	74.52 (15.34)	76.65 (13.69)					
Time						6.03	0.02*	0.03	72.50	86.25
Group						1.07	0.37	0.02	69.70	88.40
Time×Group						1.15	0.34	0.02	66.70	92.00
Mental processing index (Std scores)										
7 years	70.52 (7.96)	70.87 (7.75)	73.95 (6.20)	71.43 (7.42)	76.18 (9.93)					
9 years	72.00 (6.96)	73.67 (7.65)	73.82 (5.81)	72.96 (11.65)	76.16 (11.72)					
Time						1.81	0.18	0.01	72.16	81.84
Group						1.17	0.33	0.03	70.00	85.40
Time×Group						0.96	0.43	0.03	68.88	86.40

Data in columns 2‐6 are M (SD). ART‐40W, ART 40 weeks (treatment started early with planned interruption at 40 weeks); ART‐96W, ART 96 weeks (treatment started early with planned interruption at 96 weeks); ART‐Def, ART‐Deferred (treatment deferred until clinical or immunological disease progression was evident); CHEU, children HIV‐exposed uninfected; CHU, children HIV‐unexposed; ESE, effect size estimate (Eta‐square); KABC‐II, Kaufman Assessment Battery for Children – Second Edition.

* *p* < 0.05

** *p* < 0.01

*** *p* < 0.001.

These analyses detected no significant main effects of Group, and only one significant Time by Group interaction effect: for fine motor dexterity (PPT raw score), ART‐Def performed more poorly than ART‐40W, CHEU and CHU at the seven‐year assessment (*p* = 0.04, <0.001 and <0.001 respectively), but had the largest improvement over time with no significant pairwise differences at the nine‐year assessment (all *p*s > 0.18).

Finally, within‐group analyses detected no statistically significant effect of home language on any cognitive outcome variable at either seven or nine years of age (all *p*s > 0.08).

## 4 Discussion

With access to ART, CLWH live longer and are expected to negotiate academic, familial and social transitions into adolescence and adulthood as successfully as uninfected children. Hence, it is important to understand their cognitive development over time. Here we compared cognitive performance at seven and nine years old, of CLWH who had been randomized into early time‐limited ART (ART‐40W and ART‐96W) with planned interruption, or deferred ART initiation until clinical or immunological disease progression (ART‐Def), according to WHO 2006 ART treatment criteria.

The primary value of this study is its longitudinal design, which allowed evaluation of the effects of three ART strategies on cognitive outcomes at two time points and on the trajectory of change between measurements. Moreover, the tests were conducted across a developmentally critical period when the foundations of adult cognitive architecture are being established [32]. Other strengths are that we (a) assessed children in a narrow age band, thus effectively eliminating large age‐related variability in performance, (b) only included CLWH who initiated ART early in life, (c) drew a sample from a geographic region heavily affected by HIV and characterized by infrastructural and other resource limitations that might affect treatment access, (d) partially controlled for non‐HIV confounders with comparative data from uninfected children and (e) used culturally appropriate test instruments.

In analyses comparing the cognitive performance of CLWH and CHIV‐ at both seven and nine years, CLWH performed significantly worse on measures of executive functioning (specifically, semantic fluency) and auditory working memory. Analyses comparing ART‐Early, ART‐Def, CHEU and CHU demonstrated that better auditory working memory performance among uninfected participants was largely attributable to the CHU, who performed significantly better than all CLWH and CHEU.

These findings partially confirmed our hypothesis related to the impairing effects of HIV on cognition, and are consistent with a recent report that school‐aged CLWH receiving ART before age three years performed worse on an auditory working memory task than CHIV‐ controls [1]. Of note, even though more than 85% of these CLWH were virally suppressed at testing, HIV interfered with their neurodevelopment and affected major areas of cognitive (and, potentially, social and academic) functioning. For instance, deficits in semantic fluency performance might indicate difficulties in planning, monitoring and decision making. Furthermore, because previous research indicates that working memory scores predict reading and math achievement [33] these children may under‐achieve educationally.

Lastly, cognitive performance of ART‐40W, ART‐96W, ART‐Def, CHEU and CHU groups did not confirm hypotheses related to effects of early intervention. ART‐40W and ART‐96W participants had similar scores to ART‐Def participants at both seven and nine years on almost all outcome measures. These findings support those of Laughton *et al*. from the same cohort [15] in demonstrating that limited ART interruption with strict clinical monitoring in virally suppressed children who start treatment early does not negatively impact later cognitive outcomes. Our findings also support data from the original CHER trial, which reported that early time‐limited ART was safe [10].

All three series of two‐way mixed‐model repeated‐measures ANOVAs confirmed our hypothesis related to cognitive developmental trajectory most outcomes, participants in all groups performed better at nine years than at seven years. This cohort was assessed at a critical phase of brain growth when details of neuronal circuits are being calibrated for functions required of the adult brain [34]. Therefore, the improvement in performance for all children was not surprising.

Unexpectedly, however, scores for visual‐motor integration, attention and auditory working memory across all five groups were lower at nine years than at seven years. This apparent anomaly is probably explained by using normative tables based on North American standardization samples. Hence, at seven years the current sample of South African children was performing at a level close to that of the seven‐year‐old reference group, whereas at nine years their performance was farther away from the norm (i.e. although their developmental trajectory might still have been positive, it may have been less steep than the reference group). This result points to one of many potential hazards of using foreign normative data to judge the performance of local patients [35, 36].

An interesting finding was that, across all between‐group comparisons, there was a significant Time by Group interaction for PPT, which assesses fine motor dexterity. The essence of this result is that at seven years, ART‐Def participants performed significantly worse than the other four groups, whereas at nine years there were no significant between‐group differences (due to a rapid trajectory of improvement in the ART‐Def group). The first part of our result is consistent with Puthanakit *et al*. [17], who found that their early initiation group outperformed their deferred group on the PPT. The second part of our result is consistent with Laughton *et al*. [15] in suggesting cognitive recovery after deferred treatment.

Overall, our results suggest that CLWH, regardless of treatment strategy or degree of viral suppression, require monitoring throughout childhood as they remain at risk for cognitive deficits. Although our results differ from previous studies suggesting that deficits in visual‐motor integration, attention, visuospatial abilities, receptive language and learning abilities are core components of the cognitive profile in CLWH [37, 38]. Our findings suggest some deficits may resolve with age and are consistent with clinical, immunological and neurodevelopmental findings from previous CHER studies [10, 15].

Our study had the following limitations: first, although CLWH participants entered the CHER trial before three months of age, no data are available on whether the transmission was in utero or intra‐partum. Second, all CLWH were ART adherent at testing, with over 80% being virally suppressed. Such levels of adherence are not typical (they likely occurred in this study because of intense guidance, monitoring and clinical management), and so it remains unknown whether more cognitive deficits might be observed in less‐adherent individuals. Third, our test battery did not include direct measures of expressive vocabulary, a cognitive construct that previous studies suggest is an important component of HIV‐associated cognitive impairment in children and adolescents [39]. Fourth, as noted previously [10], the CHER trial did not include an early uninterrupted ART arm, thus limiting conclusions about the long‐term effects of this treatment strategy on cognitive, behavioural and physical outcomes. Fifth, because of no locally derived normative data; we used standardized scores based on US norms. Finally, our sample size was relatively small, hence the analyses might have been (a) insufficiently powered to detect subtle between‐group differences, and (b) not representative of the greater population of CLWH children.

## 5 Conclusions

In summary, our data suggest that neurocognitive performance for treatment groups and controls was similar for most domains by nine years of age, with improvements from seven years in all groups. Nevertheless, all ART‐treated CLWH, regardless of their treatment arm, remain at risk for cognitive deficits over early school ages. This risk is present even though these children experienced active and intense guidance, monitoring, clinical management and sustained viral suppression. Our data also suggest, however, that the nature of these cognitive deficits may change over time as neuropsychological development proceeds (e.g. we observed deficits in fine motor dexterity at seven years but not nine years).

Overall, our findings support previous studies reporting that, even after early introduction of ART treatment, cognitive deficits may persist and can become severe at school age [1, 40]. The identified deficits may have negative consequences for these children’s future learning, reasoning and adaptive functioning. Future investigations must examine possible persistence into and beyond adolescence and should address remediation goals. Boivin *et al*. [41, 42] have demonstrated that computerized cognitive rehabilitation training can improve working memory and problem‐solving skills in CLWH. They suggest that such interventions be employed as early as possible to prevent the progression of these HIV‐associated cognitive deficits.

## Competing interests

The authors declare that they have no conflicts of interest. The authors also declare that they have no relevant financial relationships to disclose.

## Authors’ contributions

MFC was one of the CHER trial’s principal investigators. He and others designed the trial and provided guidance to BL, EMM and AvdK for the design of the CHER‐Plus neuro and imaging sub‐study, within which this study is nested. KvW was responsible for the assessment of English‐ and Afrikaans‐speaking participants, scoring capturing and auditing data, interpreting results, writing up the final manuscript and approving the final manuscript as submitted. She will be the corresponding author and is responsible for submission. BL provided on‐site supervision to KvW throughout this study, gave clinical guidance/care for participants, reviewed the manuscript and suggested relevant changes prior to submission. MFC reviewed the manuscript and made relevant changes prior to submission. MJB provided expert advice for the design of the CHER‐Plus Neuro sub‐study, reviewed the manuscript and made relevant changes prior to submission. EMM and AvdK reviewed the manuscript and made relevant changes prior to submission. MK was responsible for statistical analysis of data, reviewed the manuscript and made relevant changes prior to submission. KGFT provided supervision of this work throughout the entire process, critically reviewed the manuscript and made relevant changes prior to submission. KGFT gave final approval of the manuscript as submitted to the journal. All authors approve the final manuscript as submitted and agree to be accountable for all aspects of the work.
